# A Case of Urethral Carcinoma With Cardiac Metastasis and Pulmonary Tumor Thrombotic Microangiopathy Confirmed by Autopsy

**DOI:** 10.7759/cureus.105220

**Published:** 2026-03-14

**Authors:** Heisuke Iijima, Makoto Obayashi, Masakatsu Ueda, Koji Yoshimura

**Affiliations:** 1 Department of Urology, Shizuoka General Hospital, Shizuoka, JPN; 2 Department of Pathology, Shizuoka General Hospital, Shizuoka, JPN

**Keywords:** autopsy, cardiac metastasis, pulmonary tumor thrombotic microangiopathy, urethral carcinoma, urothelial carcinoma

## Abstract

Cardiac metastasis and pulmonary tumor thrombotic microangiopathy (PTTM) are uncommon, often fatal complications of malignancy, and are rarely reported in urothelial carcinoma. We report an autopsy-confirmed case of a primary urethral carcinoma in a female patient that progressed into both cardiac metastasis and PTTM.

A 70-year-old woman complaining of a palpable urethral mass was referred to our urology department during treatment for an infected hip prosthesis. Her history included stage IIIB cervical cancer treated with pelvic radiotherapy, followed by long-term intermittent self-catheterization for neurogenic bladder. Imaging demonstrated a urethral tumor without distant metastases, and initial needle biopsy suggested squamous cell carcinoma. Multimodal therapy was initially considered but limited by infection and renal dysfunction. Local radiotherapy achieved initial tumor reduction, and she subsequently underwent anterior pelvic exenteration with right nephroureterectomy for a severely atrophic right kidney. Pathology revealed invasive urothelial carcinoma with squamous differentiation and positive pelvic lymph nodes. Four months post-surgery, she developed lower limb edema and deep vein thrombosis. Echocardiography revealed a right ventricular mass, initially suspected to be infective vegetation or tumor thrombus. Treatment was limited by bleeding risk, and the cardiac mass progressed, ultimately leading to death.

Autopsy disclosed a large tumor nearly filling the right heart chambers, histologically consistent with metastatic urothelial carcinoma. Microscopic examination of the lungs revealed tumor emboli with intimal proliferation and thrombosis consistent with PTTM.

We present a rare case of primary urethral carcinoma complicated by both cardiac metastasis and PTTM, confirmed by autopsy. This report highlights aggressive disease behavior, diagnostic challenges, and the importance of considering uncommon metastatic patterns in advanced urothelial malignancies.

## Introduction

Cardiac metastasis from urothelial carcinoma is rare [1,2]. To the best of our knowledge, cardiac metastasis originating specifically from urethral carcinoma has only been reported once [3]. Additionally, pulmonary tumor thrombotic microangiopathy (PTTM) secondary to urological cancers is also extremely rare [4]. Here, we report a unique case of invasive urethral carcinoma that developed both cardiac metastasis and PTTM, confirmed by autopsy.

## Case presentation

A 70-year-old woman was referred to our urology department for complaints of a urethral mass during hospitalization for treatment of an infected left hip prosthesis. Her medical history was notable for stage IIIB cervical cancer at age 36 years, treated with pan-pelvic radiotherapy (50 Gy in 25 fractions), which was followed by long-term intermittent self-catheterization for neurogenic bladder. She had also undergone left hip arthroplasty in her late 60s following a left femoral fracture.

**Figure 1:**
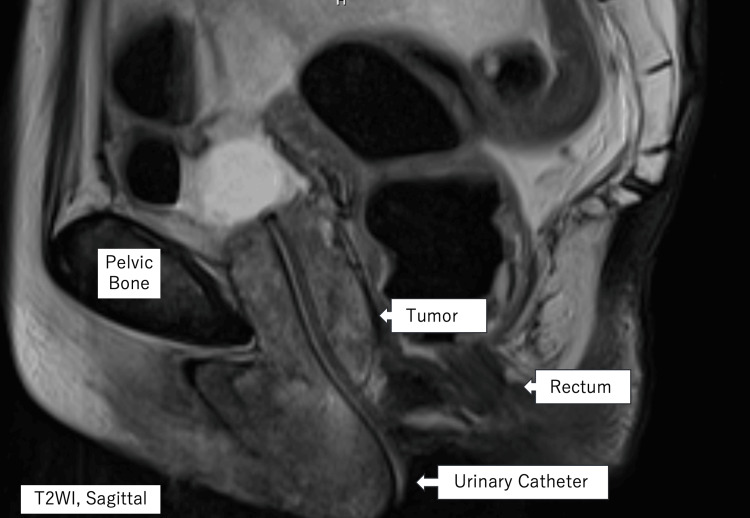
Pelvic MRI at initial presentation A T2-weighted sagittal MRI of the pelvis shows an oval-shaped tumor occupying the entire length of the urethra.

On physical examination, a painless tumor was palpable at the urethral meatus. MRI revealed a 25 × 35 × 45 mm mass extending along the entire length of the urethra (Figure 1).

No distant metastases were detected on CT. The right kidney showed marked hydronephrosis and atrophy for reasons unknown. 

Laboratory tests showed anemia (hemoglobin: 8.2 g/dL), impaired renal function (creatinine: 2.5 mg/dL), and elevated serum tumor markers (squamous cell carcinoma antigen: 6.3 ng/mL and cytokeratin 19 fragment (CYFRA 21-1): 4.2 ng/mL) (Table 1). 

**Table 1:** Laboratory findings at initial presentation CYFRA, cytokeratin 19 fragment.

Parameter	Value	Unit	Reference range
White blood cell count	5.5	×10⁹/L	3.3-8.6
Hemoglobin	8.2	g/dL	11.6-14.8
Platelet count	177	×10⁹/L	158-348
Serum creatinine	2.5	mg/dL	0.46-0.79
Estimated glomerular filtration rate	16	mL/min/1.73 m²	≥90
Sodium	138	mmol/L	138-145
Potassium	4.3	mmol/L	3.6-4.8
Chloride	109	mmol/L	101-108
Squamous cell carcinoma antigen	6.3	ng/mL	<1.5
CYFRA 21-1	4.2	ng/mL	<3.5

**Figure 2:**
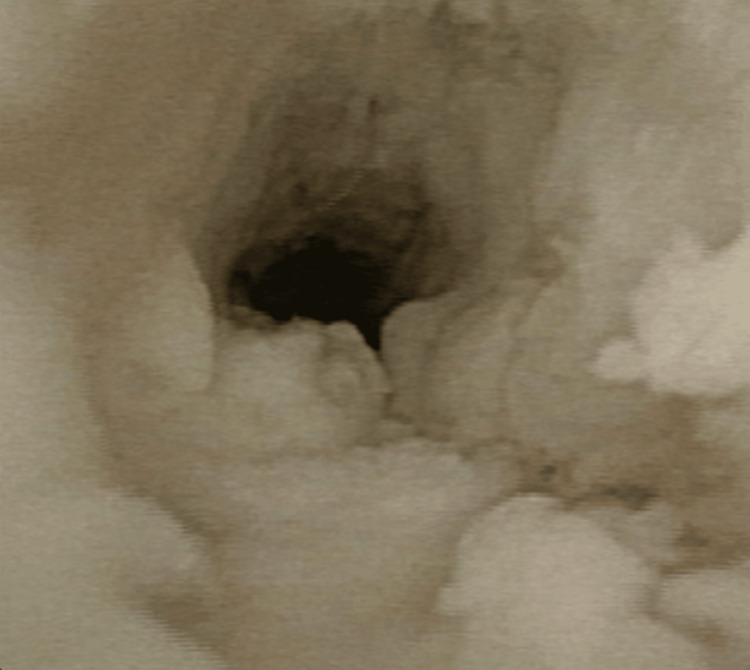
Cystoscopic image of the urethra The entire length of the urethra appears white and filled with necrotic tissue.

On cystoscopy, necrotic-appearing mucosa extended continuously along the entire length of the urethra (Figure 2).

Given the extent and appearance of the lesion, endoscopic sampling was considered unlikely to provide an adequate diagnostic specimen, and transurethral biopsy under anesthesia was considered impractical due to the patient’s active prosthetic joint infection and overall clinical condition. A transvaginal needle biopsy of the lesion confirmed moderately differentiated squamous cell carcinoma. She was therefore clinically diagnosed with urethral carcinoma, cT3N0M0, although the initial biopsy findings were limited and did not fully reflect the final pathological diagnosis. 

**Figure 3:**
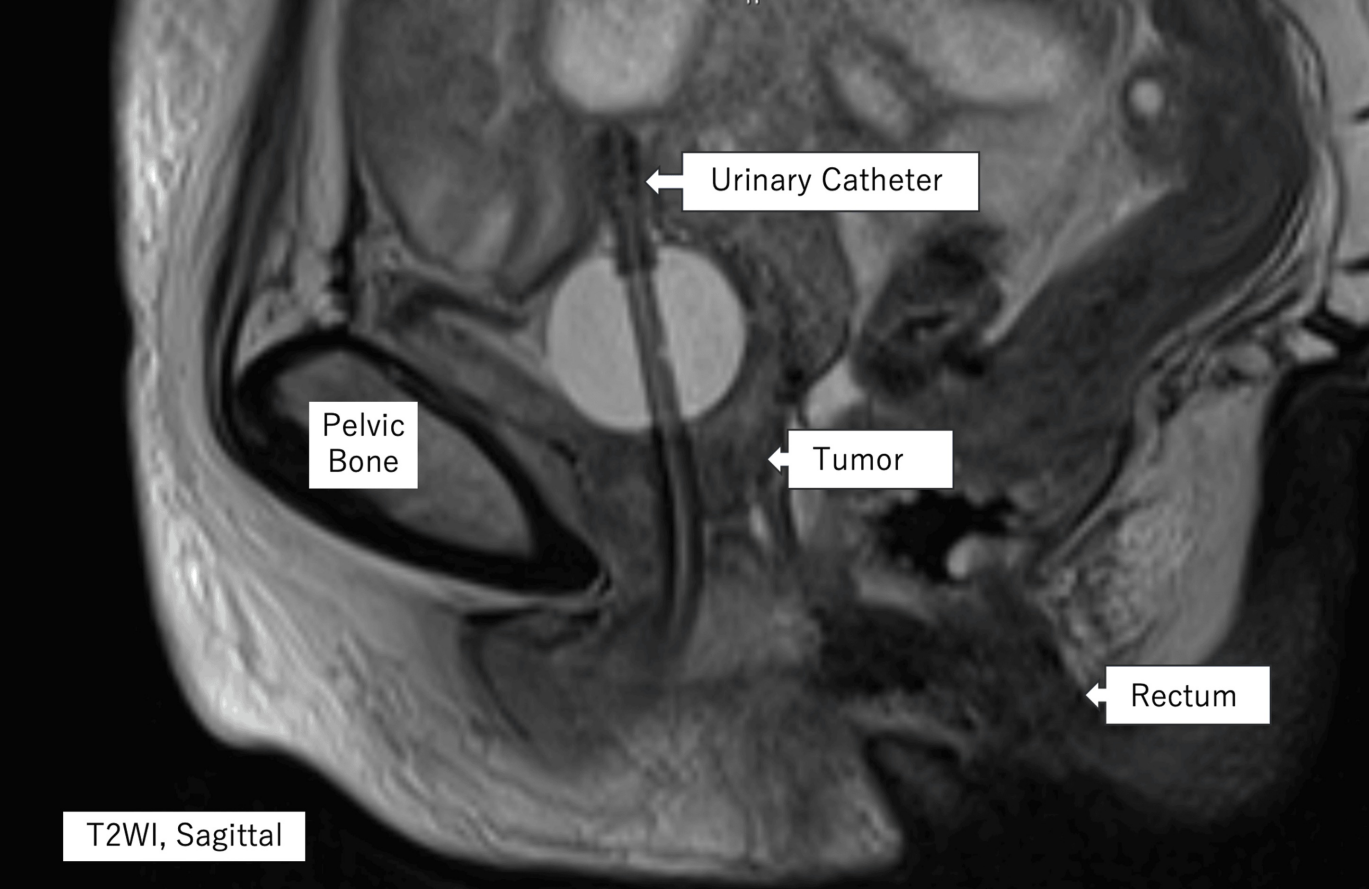
Pelvic MRI after radiotherapy A T2-weighted sagittal image of the pelvis taken post-radiotherapy shows marked tumor regression.

A multimodal approach of chemoradiotherapy with platinum-based cytotoxic agents, in combination with surgery, was initially planned. However, her concurrent prosthetic infection and impaired renal function precluded chemotherapy and surgery. As an alternative, she underwent radiotherapy to the tumor (60 Gy in 30 fractions) while she was treated for her infection, despite her previous pelvic irradiation. The radiation dose was selected after careful multidisciplinary discussion, balancing oncologic control against the increased risk associated with prior pelvic irradiation. Follow-up MRI three months later showed approximately a 50% reduction in tumor size (19 × 19 × 23 mm) (Figure 3).

As her infection control was not readily achieved, she was finally deemed fit for surgery after six months of antibiotic treatment.

Given her prior pelvic radiotherapy for cervical and urethral cancer, the surgical risk was considered relatively high. An anterior pelvic exenteration, right nephroureterectomy, and left cutaneous ureterostomy were performed. The surgery was performed in the supine position via a midline abdominal incision. Dense adhesions between the intestines and peritoneum were encountered. Right nephroureterectomy was performed first, as minimal residual right kidney function was expected. This was followed by cystectomy and hysterectomy. The cervix and vaginal tissues were markedly atrophic due to prior radiation. After exenteration, the labia were sutured closed, and a left cutaneous ureterostomy was constructed using the Toyoda method [5]. Pelvic lymph node dissection was also performed. The operation lasted 9 hours and 48 minutes, with 2060 mL of blood loss.

**Figure 4:**
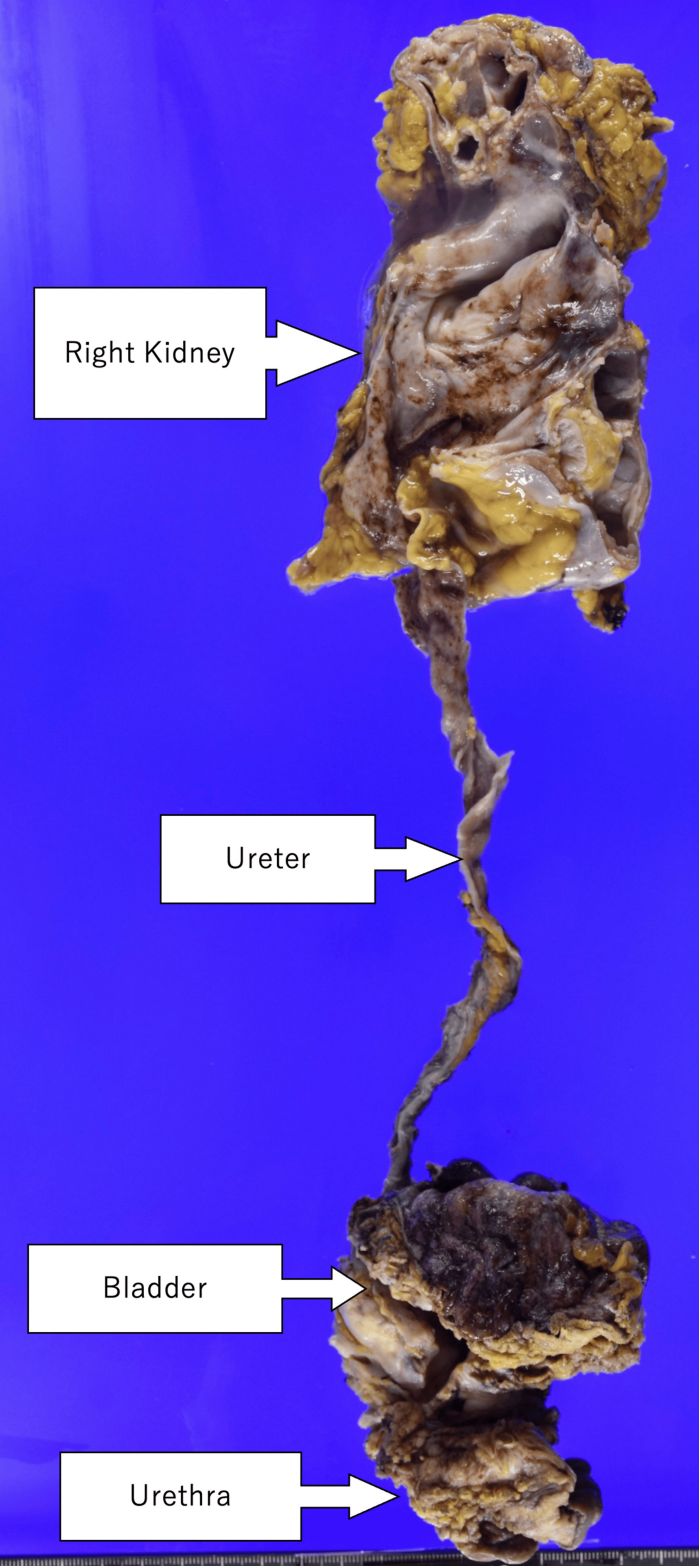
Surgical specimen The bladder, urethra, right kidney, and right ureter are removed en bloc.

**Figure 5:**
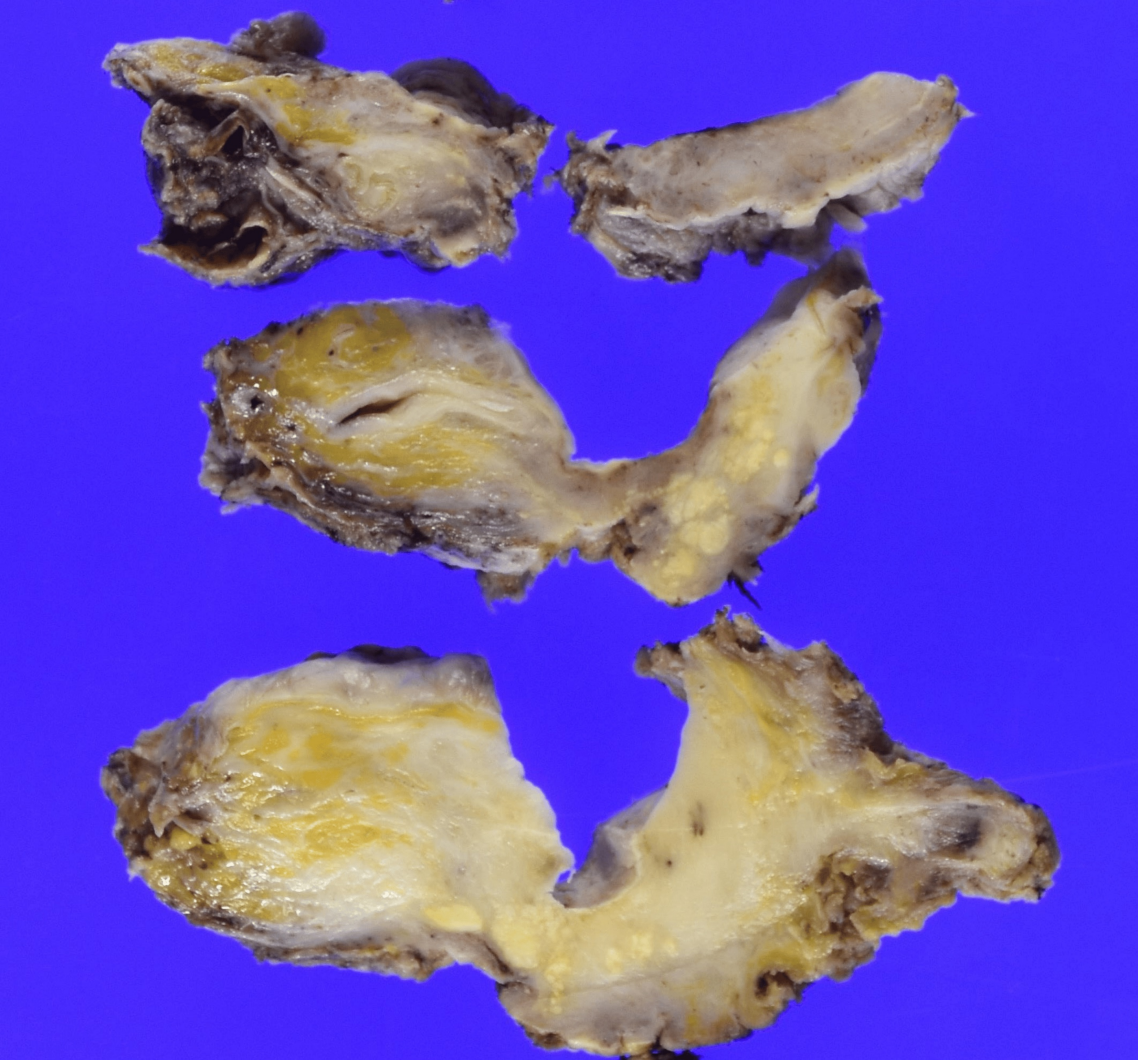
Cross-section of the bladder A cross-section from the bladder to the urethra shows a wall that appears white and thickened.

**Figure 6:**
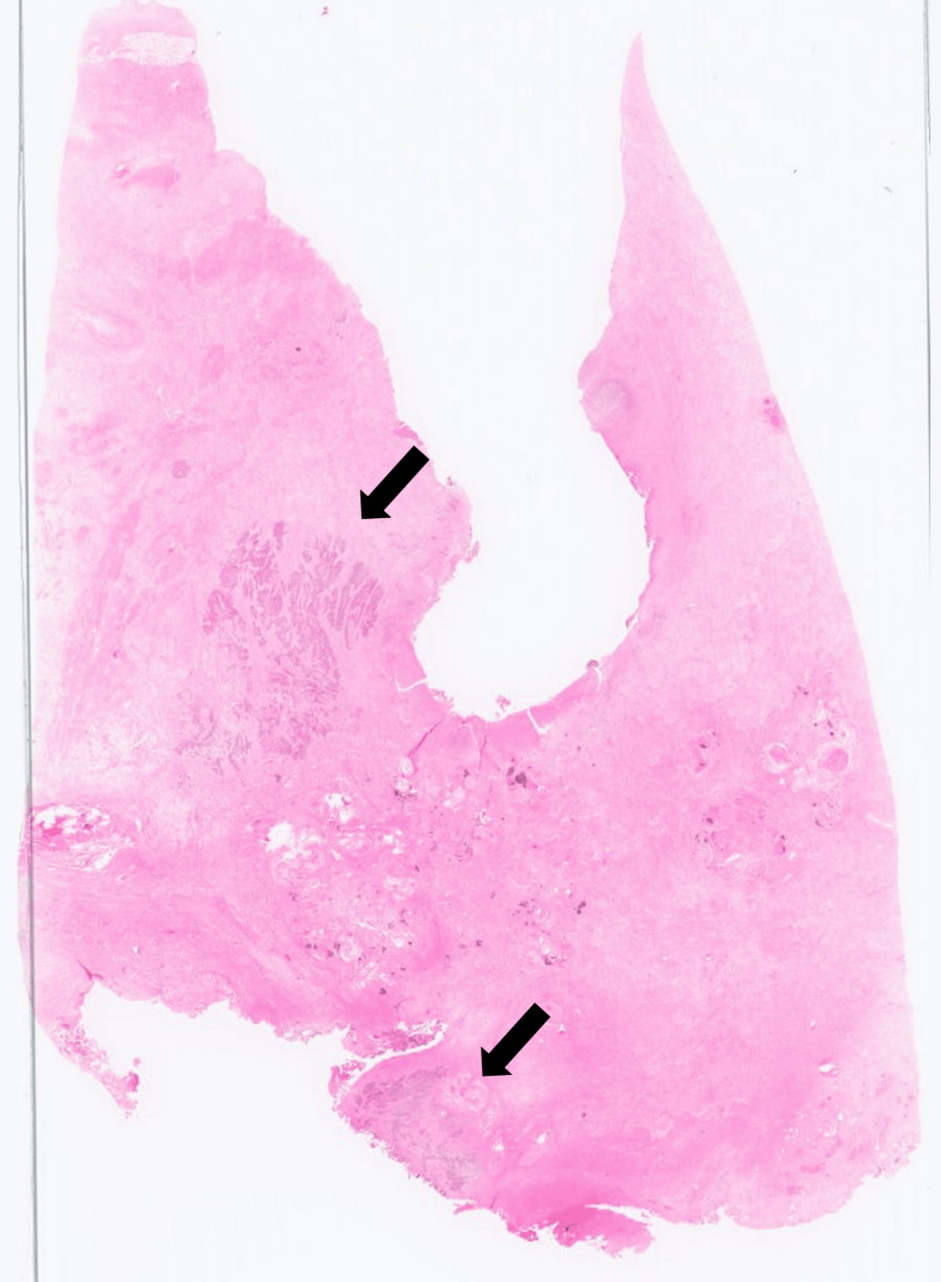
Cross-section H&E stain of the urethra A magnified view of the urethral segment reveals that most of the tumor has regressed, with a few viable tumor foci remaining (arrow). H&E, hematoxylin and eosin.

**Figure 7:**
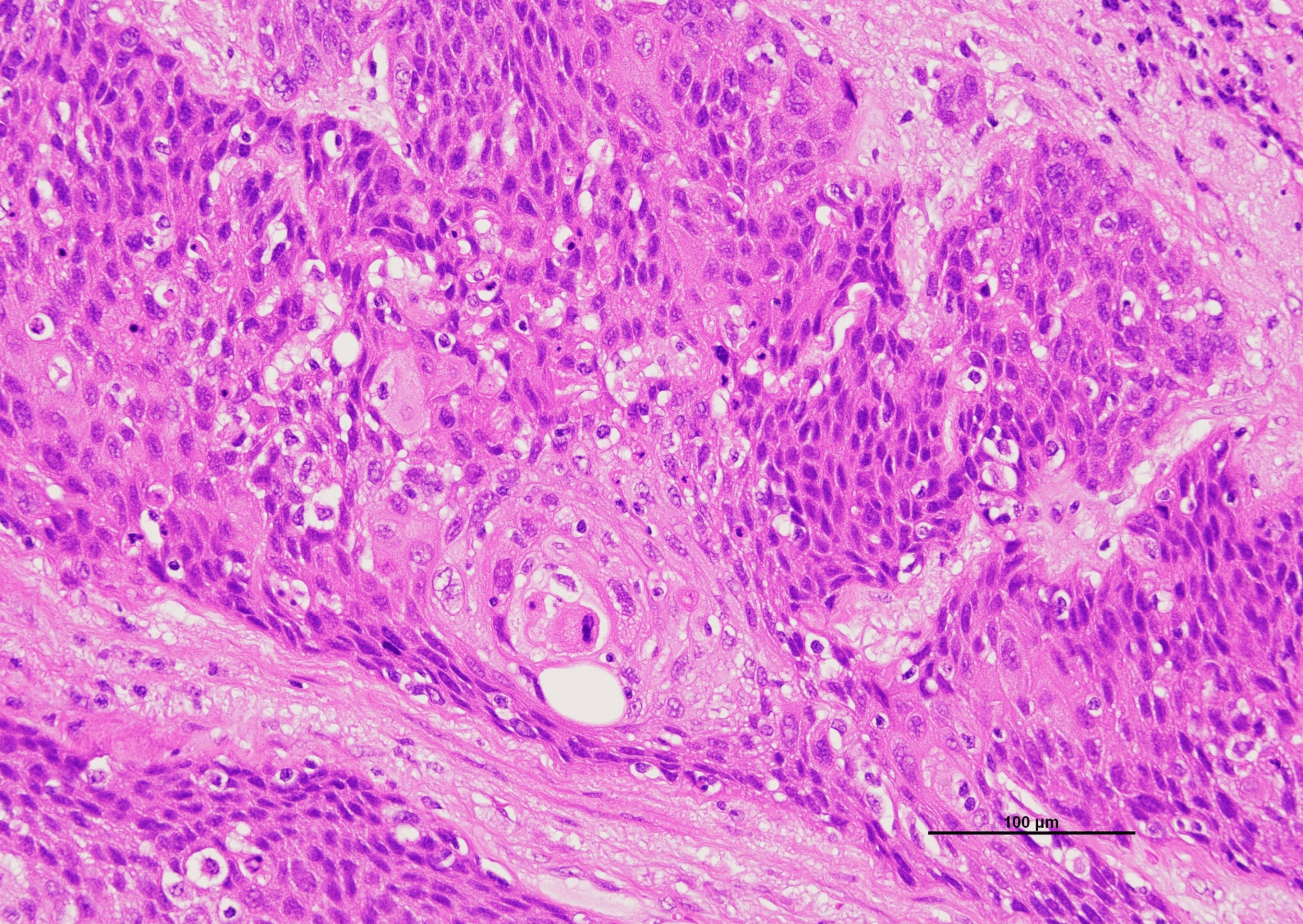
Magnified view of the urethral tumor, H&E stain Invasive urothelial carcinoma with squamous differentiation is observed within the urethral wall (×200). H&E, hematoxylin and eosin.

Pathological analysis confirmed urothelial carcinoma of the urethra with squamous differentiation (pT4N1M0) (Figures 4-7), with a positive surgical margin and metastasis in the left external iliac lymph node.

Postoperatively, the patient developed paralytic ileus but gradually recovered and was discharged on post-operative day 17 without further complications.

Adjuvant immunotherapy with nivolumab was initiated based on the final pathology and available evidence supporting its use after radical surgery in urothelial carcinoma [6]. However, this was discontinued after only one cycle due to Grade 3 immune-related hepatitis.

Four months post-surgery, the patient developed progressive bilateral lower limb edema. Ultrasound revealed deep vein thrombosis (DVT) in the popliteal veins. Transthoracic echocardiography identified a mass in the right ventricle, raising suspicion for either infective endocarditis or cardiac metastasis. Shortly after anticoagulation therapy was initiated, the patient experienced progressive anemia and melena. Colonoscopy suggested intestinal hemorrhage likely exacerbated by anticoagulation in the setting of prior pelvic irradiation. Given the bleeding risk and her overall frailty, further treatment of the venous thrombosis and cardiac mass was not possible. Blood cultures were negative, supporting a non-infectious etiology. The cardiac mass gradually increased in size, and pleural effusions developed approximately five months after detection of the cardiac mass. She died of suspected acute right heart failure several months after the diagnosis of cardiac metastasis. An autopsy was performed with family consent.

**Figure 8:**
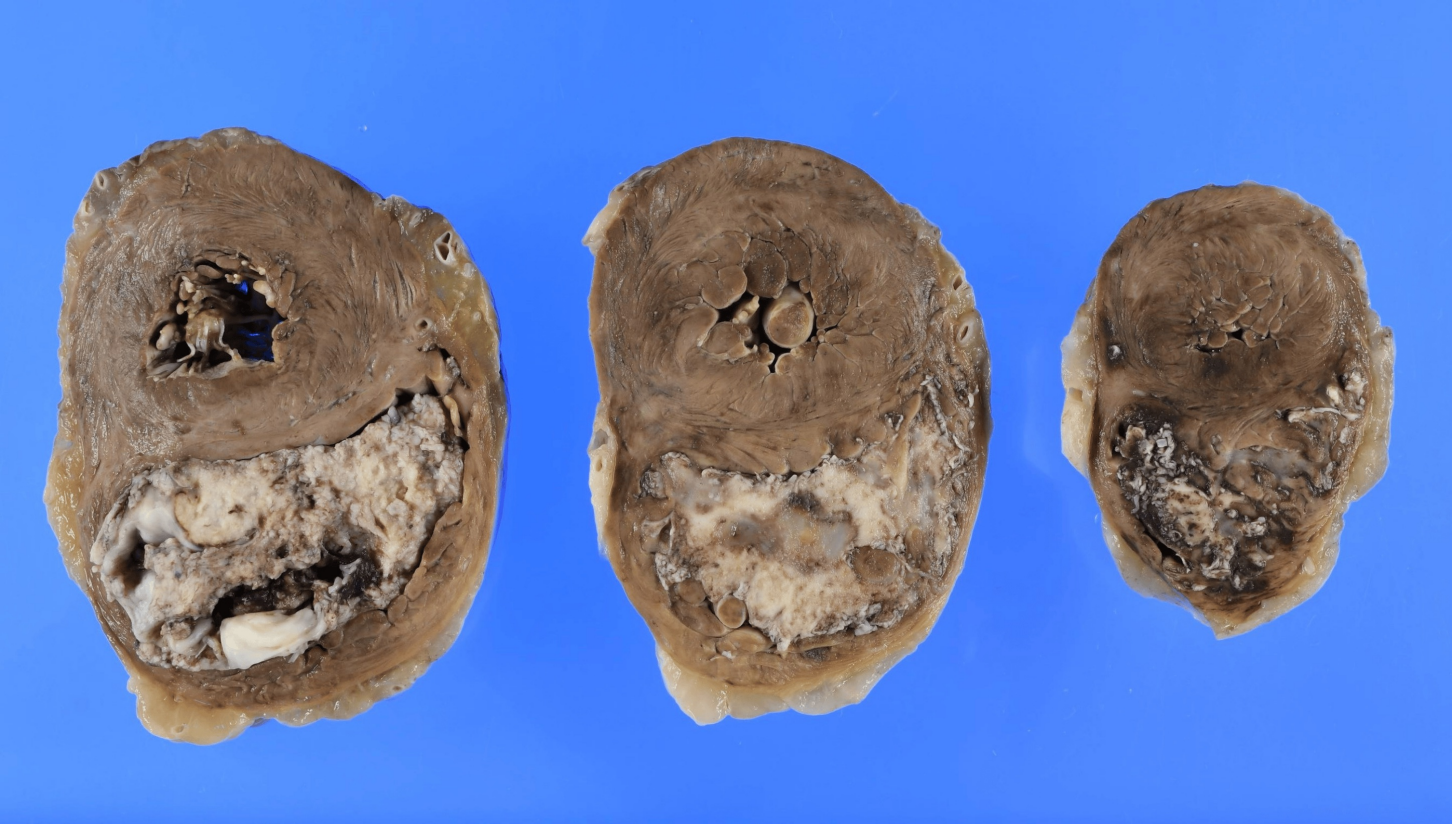
Gross autopsy findings of right-sided cardiac metastasis The heart after fixation shows the lumen of the right ventricle filled with a white, solid tumor.

**Figure 9:**
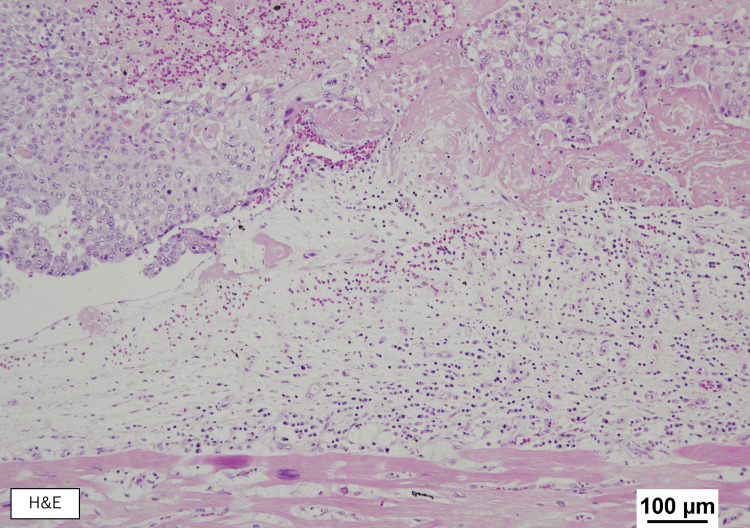
Histologic autopsy findings of right-sided cardiac metastasis The tumor (shown in the top half of the image) is observed infiltrating the endocardium (bottom half of the image). H&E, hematoxylin and eosin.

At autopsy, the patient's height was 150 cm and weight was 49.2 kg. No local recurrence of cancer was detected. However, metastases were present in the heart, lungs, and para-aortic lymph nodes. A 60 mm necrotic tumor occupied almost all the right heart chambers, with invasion into the endocardium and associated ventricular hypertrophy (Figures 8, 9).

**Figure 10:**
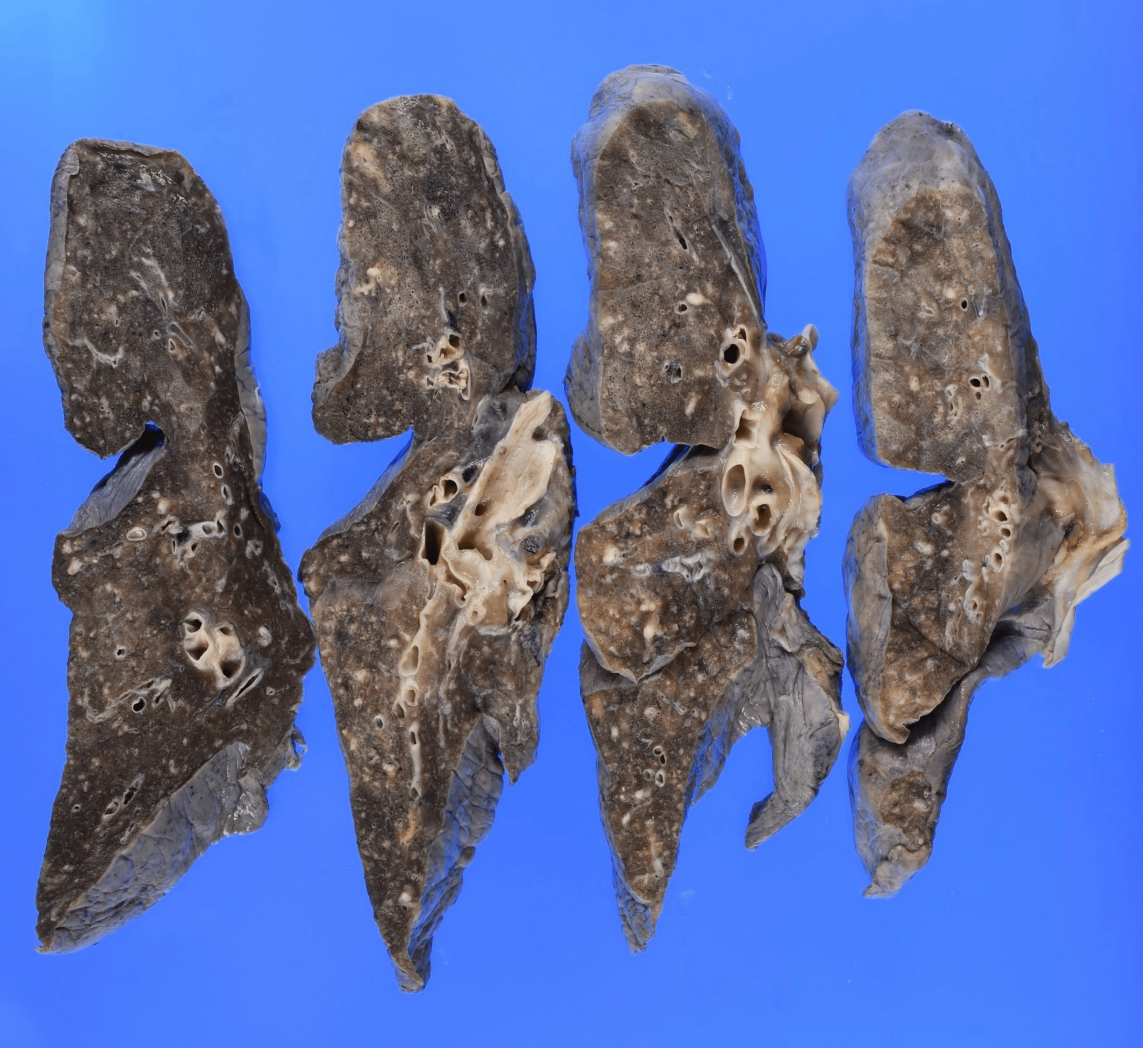
Gross autopsy findings of PTTM In the fixed right lung, tumor emboli within the pulmonary arteries are observed as white micronodules. PTTM, pulmonary tumor thrombotic microangiopathy.

The tumor in the right ventricular cavity was identified as urothelial carcinoma with squamous differentiation, exhibiting the same histological features as the urethral tumor. In both lungs, tumor emboli within the pulmonary arteries were observed as white micronodules (Figure 10).

**Figure 11:**
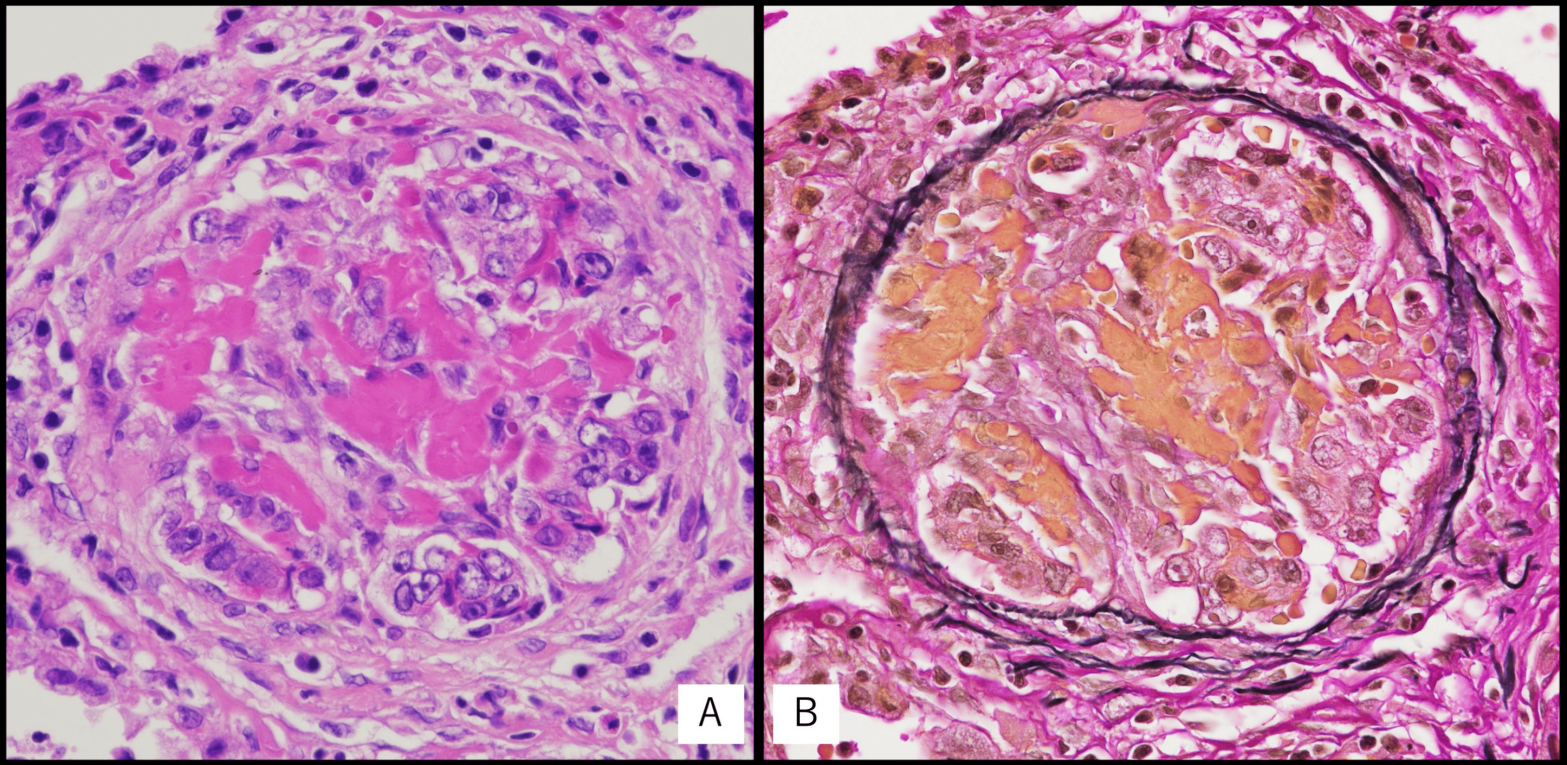
Histologic autopsy findings of PTTM A. Microtumor emboli, recanalization of several thrombi, and luminal narrowing are observed in minute vessels with a diameter of 120 μm (×200). B. EVG staining of the same region shown in Figure 11A reveals thrombus organization (×200). EVG, Elastica van Gieson; PTTM, pulmonary tumor thrombotic microangiopathy.

Microscopically, microtumor emboli, organized thrombi, and luminal narrowing were observed in the minute vessels, consistent with PTTM (Figure 11).

Two metastatic para-aortic lymph nodes were identified. Additionally, radiation-induced angiopathy was observed in the small intestine, sigmoid colon, and rectum, with hyalinized arterioles and capillaries. Intraluminal blood was also present. The liver showed perivenular fibrosis, consistent with her history of immune-related hepatitis. Bilateral pitting edema was prominent, and severe pleural effusions were noted (850 mL on the right and 400 mL on the left). Finally, the cause of death was attributed to cardiopulmonary failure driven by extensive cardiac metastasis and PTTM in the setting of metastatic urothelial carcinoma. 

## Discussion

Urethral carcinoma is a rare malignancy, accounting for less than 1% of all genitourinary cancers, with common histological subtypes, including urothelial carcinoma, adenocarcinoma, and squamous carcinomas [7,8]. In women, risk factors for urethral carcinoma include chronic inflammation, long-term catheterization, recurrent urinary tract infections, high-risk human papillomavirus infection, and a history of pelvic radiotherapy [7,8]. These factors are thought to contribute to metaplastic and dysplastic changes in the urethral epithelium, increasing susceptibility to malignant transformation. There is currently no standardized treatment strategy for advanced urethral carcinoma (T3-T4), and such cases are often managed with a multimodal approach that may include radical surgery, radiotherapy, and platinum-based chemotherapy [7-9]. Prognosis is strongly influenced by nodal involvement and distant metastasis [7,8].

In our patient, prior pelvic radiotherapy, neurogenic bladder with long-term intermittent self-catheterization, and chronic inflammation may have contributed to the development of urethral cancer. In retrospect, the initial biopsy appears to have sampled a region enriched in squamous differentiation, which likely accounted for the discrepancy between the initial diagnosis of squamous cell carcinoma and the final pathology of urothelial carcinoma with squamous differentiation. Her treatment strategy was complicated by active infection of her prosthetic joint and underlying renal dysfunction, precluding the use of neoadjuvant platinum-based chemotherapy. Radiotherapy was used as first-line therapy despite prior pelvic irradiation due to limited therapeutic options. Although the tumor showed a favorable initial response, systemic progression occurred rapidly following radical surgery and short-term immunotherapy with nivolumab. The rapid deterioration after immunotherapy raises the possibility of immune checkpoint inhibitor-associated hyperprogression [10], although this remains speculative.

Cardiac metastasis is relatively common in autopsy studies, occurring in up to 18% of patients with cancer [1,2]. However, it is rarely diagnosed antemortem [2]. Among urological cancers, renal cell carcinoma is the most frequently associated with cardiac metastasis, and urothelial carcinoma is rare [1]. To our knowledge, this is the second reported case of cardiac metastasis from urethral carcinoma, following a report of a male case by Brehm et al. in 2010 [3]. In contrast to the isolated cardiac metastasis reported by Brehm et al., the present case demonstrated rapidly progressive and widespread disease, including PTTM and multiple cardiac metastases, despite the presence of squamous components in both cases. Tumor spread to the heart typically occurs via hematogenous or lymphatic pathways and is usually initially asymptomatic [1,2]. With disease progression, patients may present with heart failure, arrhythmia, or thromboembolism [1,2]. Antemortem diagnosis is challenging and often requires high clinical suspicion [1]. While transthoracic and transesophageal echocardiography may detect intracardiac lesions, definitive diagnosis requires histologic confirmation through biopsy or surgical excision [11]. Treatment options vary from surgical excision to systemic therapy of the underlying cancer.

**Figure 12:**
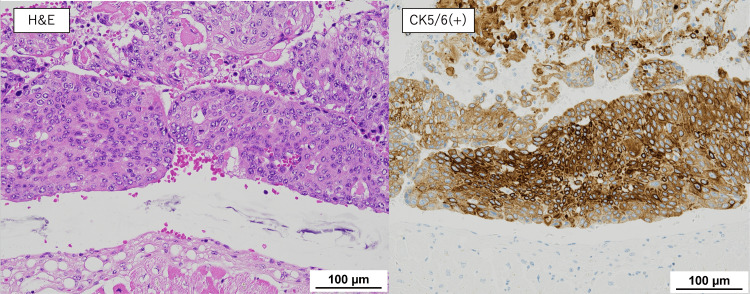
Histological analysis of the cardiac tumor The cardiac tumor showed positive staining for CK5/6, consistent with that of the primary tumor. H&E, hematoxylin and eosin.

In our patient, cardiac metastasis was identified incidentally, following the diagnosis of DVT, causing bilateral lower limb edema. The right ventricular mass was initially presumed to be either vegetation from infectious endocarditis or metastasis, though a negative blood culture and its progressively growing size raised suspicion for tumor thrombus. Autopsy confirmed massive tumor volume, occupying much of the right heart chambers. Endocardial infiltration was visible, though limited only at a superficial level. No myocardial or pericardial involvement was observed. We therefore speculate a hematogenous spread of the tumor from the primary site, initially manifesting in the right heart chambers as tumor emboli, eventually fixing itself with the heart by infiltrating the endocardium. Although the primary tumor was urothelial carcinoma, squamous differentiation may partly explain the aggressive clinical course, including cardiac metastasis. On immunohistochemistry, the tumor stained positive for CK5/6 and negative for p16, p40, p63, CK7, CK20, GATA-3, and uroplakin II, which was consistent with the primary tumor (Figure 12).

In addition, we were able to rule out metastasis from cervical cancer, as immunostaining from the cervical tissue stained positive for p16 and p40. 

PTTM is a rare and often fatal form of tumor-related pulmonary hypertension. PTTM was first described by von Herbay in 1990 and is most frequently associated with gastric signet-ring cell carcinoma [12]. It is characterized histologically by widespread microscopic tumor emboli in pulmonary arterioles, accompanied by fibrocellular intimal proliferation and thrombosis, leading to progressive pulmonary hypertension and right heart failure [4,12]. Similarly to cardiac metastasis, antemortem diagnosis is difficult, as imaging findings are often non-specific. In rare cases, pulmonary artery catheter biopsy or transbronchial lung biopsy has achieved antemortem diagnosis [13]. Prognosis is extremely poor, with a rapidly deteriorating clinical course, often progressing over days to weeks once symptoms develop [4,12,14]. To our knowledge, this is the first autopsy-confirmed case of PTTM secondary to urethral carcinoma.

In our patient, the presence of rapidly progressive dyspnea, pleural effusions, and right heart strain in the context of cardiac tumor burden suggested possible PTTM, but diagnosis was not established during life. Autopsy confirmed both PTTM and cardiac metastasis, which likely contributed synergistically to right heart failure and respiratory failure. The mechanism of clinical deterioration was likely multifactorial, including progressive intracardiac tumor burden causing functional obstruction, PTTM leading to pulmonary hypertension, and subsequent right heart failure. Arrhythmia could not be excluded, although it was not documented clinically.

This case highlights the diagnostic challenges of rare metastatic syndromes and underscores the importance of considering such complications in patients with advanced urothelial malignancy and unexplained cardiopulmonary symptoms.

## Conclusions

We report a rare case of female urethral carcinoma complicated by both cardiac metastasis and PTTM, confirmed by autopsy. This case highlights the aggressive nature of advanced urethral carcinoma and underscores the diagnostic and therapeutic challenges posed by rare metastatic patterns such as PTTM and intracardiac tumor invasion. Early recognition and multidisciplinary decision-making are essential, especially in patients with complex comorbidities and treatment limitations.
